# Non-syndromic Association of Natal Teeth in a Case of Septic Arthritis: Does Correlation Imply Causality?

**DOI:** 10.7759/cureus.27061

**Published:** 2022-07-20

**Authors:** Kavita Hotwani, Nilima R Thosar

**Affiliations:** 1 Pediatric and Preventive Dentistry, Vidya Shikshan Prasarak Mandals (VSPM) Dental College and Research Centre, Nagpur, IND; 2 Pediatric and Preventive Dentistry, Sharard Pawar Dental College and Hospital, Wardha, IND

**Keywords:** correlation, case, septic arthritis, sepsis, natal teeth

## Abstract

Disturbances of dental development may result in anomalies, which may be apparent as soon as the child is born. This report aims to describe the occurrence of natal teeth in a non-syndromic case of neonatal septic arthritis of the knee joint. Various systemic conditions have been associated with the occurrence of natal teeth in the past. The present report highlights the importance of a proper referral system between the pediatrician and the pediatric dentist to provide a multidisciplinary approach in the first few months of life.

## Introduction

In human dentition, a normal eruption occurs by the axial movement of a tooth from its non-functional, developmental position in the alveolar bone to a functional position of occlusion. However, significant deviations from accepted norms of eruption are often observed in clinical practice [1]. In some cases, teeth appear prematurely in an infant’s oral cavity. Premature tooth eruption might be a harbinger of a systemic condition or an indication of altered physiology of the craniofacial complex. Several terms have been used, namely fetal teeth, predeciduous teeth, and dentitia praecox to designate these teeth present at the time of birth or which erupt during the first month of life. Massler et al. defined natal teeth as teeth that are present at the time of birth and neonatal teeth as those that erupt within the first 30 days of life [2].

As very few case reports of natal teeth were published in the past, this anomaly was associated with many myths [3]. The incidence of natal teeth is approximately 1:2,000 to 1:3,000 live births [4]. They can cause feeding problems, ulceration of the tongue, and may pose a risk of aspiration. Natal teeth are associated with many systemic and genetic conditions [5]. We report here an interesting case of natal teeth in a neonate with septic arthritis.

## Case presentation

**Figure 1:**
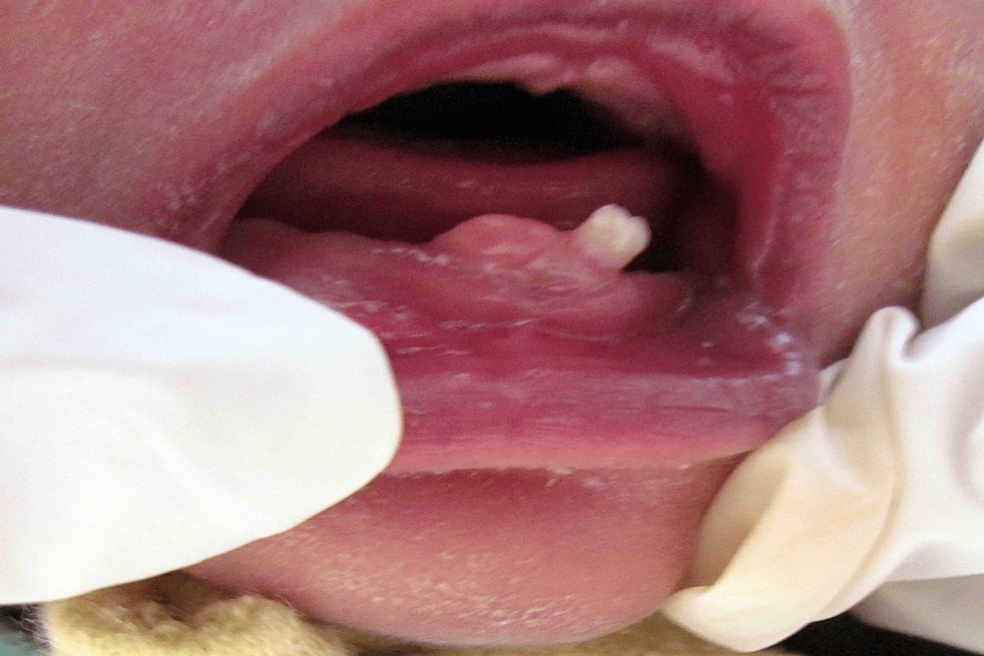
Intraoral examination showing natal tooth

A 22 -day-old male neonate was referred to the department of pedodontics by his attending pediatrician for the presence of a tooth in the lower jaw since birth and difficulty in feeding. The patient’s mother reported the presence of two teeth in the oral cavity since birth; the right erupted tooth exfoliated four days after birth during feeding as narrated by the mother. On clinical examination of the child, a single mandibular tooth crown was seen in the anterior region. It was whitish and opaque in appearance and exhibited grade I mobility. A diagnosis of the natal tooth was made. The crown size was normal and it was attached to a small pad of soft tissue above the alveolar ridge. No other lesions were observed on the tongue and associated areas (Figure 1).

**Figure 2:**
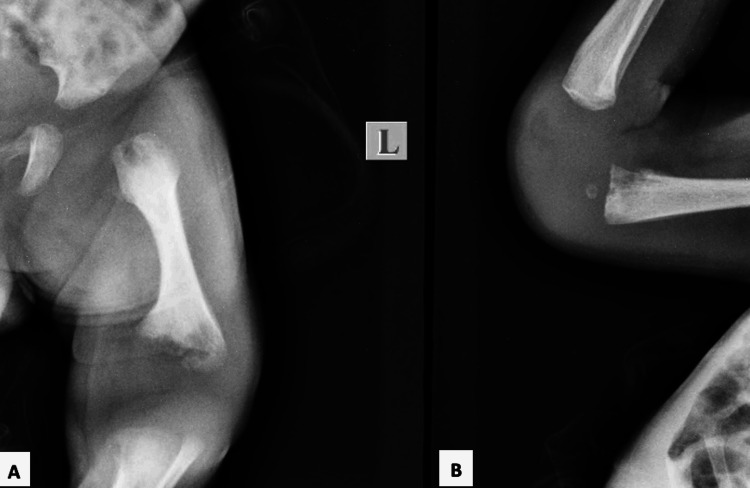
Radiograph depicting widening of joint space with the left knee joint (A) and normal elbow joint (B)

Medical history revealed that the child, born after 34 weeks of gestation, weighed 1.9 kg after cesarean delivery. His maternal history did not reveal any medication use during pregnancy. There was no familial history of similar complaints. The parents reported to the pediatrician with the complaint of the inability of the child to move both lower limbs since birth and continuous crying while attempting to move limbs. Clinical examination revealed swelling present over the left knee joint which was tender on palpation, the temperature was raised and restriction of movements observed. Radiological examination of the left knee and elbow joint was carried out which showed widening of joint space indicative of effusion with the knee joint (Figure 2, A). The elbow joint was found normal (Figure 2, B). On ultrasound examination, the presence of joint effusion was confirmed. In addition, a septic screen was conducted which revealed positive C-reactive protein indicative of sepsis and raised erythrocyte sedimentation rate. A short systolic physiologic murmur was also present on cardiovascular examination, however, there was no associated congenital cardiovascular malformation. Based on complete clinical examination, radiological findings, and blood investigations, a diagnosis of non-syndromic septic arthritis with the left knee joint was made. Joint aspiration was carried out and culture revealed *Klebsiella* and *Pseudomonas* as the offending organisms. Antibiotic therapy with cephalosporin and aminoglycoside was initiated along with analgesics, and drainage of the joint was performed followed by joint splinting for two weeks as a conservative approach. 

**Figure 3:**
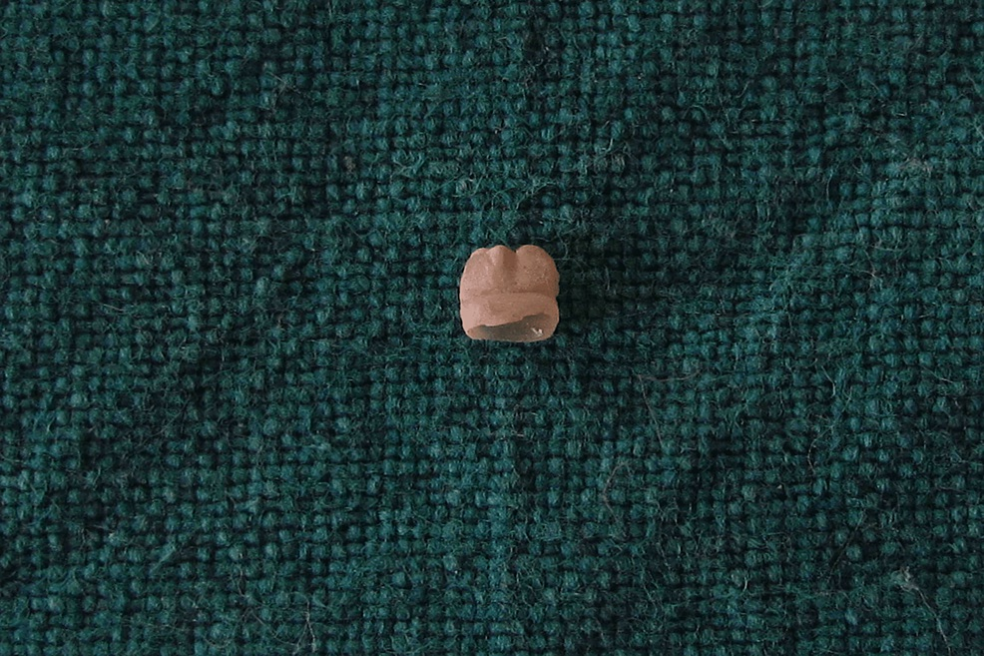
Natal tooth showing shell appearance

The child was referred for extraction of the tooth due to difficulty in feeding and the risk of aspiration. The pediatrician was consulted regarding vitamin K administration to the infant and it was found that prophylactic administration had already been done. The natal tooth was extracted under local anesthesia (lignocaine with epinephrine) using an insulin syringe. The extracted tooth had a crown but was devoid of root (Figure 3). The infant was re-evaluated after two days, and the recovery was uneventful.

## Discussion

Natal teeth are predeciduous teeth with hornified epithelial structures without roots. They may occur on the gingiva over the crest of the ridge and may be easily removed. These teeth may arise from an accessory bud of the dental lamina ahead of the deciduous bud [6]. It has been reported that 85% of natal and neonatal teeth erupt in the mandibular anterior region which was also the presentation in our case [7].

Clinically, natal teeth are small or of normal size, conical or of normal shape. Hebling et al. [8] classified natal tooth based on the appearance into four categories as a shell-shaped crown, solid crown, an eruption of the incisal margin of the crown, or edema of gingival tissue with an unerupted but palpable tooth. In the present case, the extracted natal tooth was shell-shaped and without root formation. These teeth are also generally said to be attached to a pad of soft tissue above the alveolar ridge which was evident in the present case. 

The occurrence of natal and neonatal teeth is a biological disturbance with unknown etiology. Various etiological factors have been implicated previously with natal teeth such as the superficial position of the germ, infection or malnutrition, febrile states, eruption accelerated by febrile incidents or hormonal stimulation, hereditary transmission of a dominant autosomal gene, osteoblastic activity inside the germ area related to the remodeling phenomenon and hypovitaminosis. There is no conclusive evidence of a correlation between early eruption and any systemic condition or syndrome. Some investigators, however, suggest that natal teeth may be associated with some syndromes such as Hallerman-Streiff, Ellis-Van Creveld, craniofacial dysostosis, multiple steacystoma, congenital pachyonychia, and Sotos syndrome [3].

The most acceptable theory of the presence of natal teeth is based upon the result of a superficial localization of the dental follicles, probably related to a hereditary factor. But it is necessary to investigate the possible local or systemic factors that could be related to the eruption of natal teeth and their association with other pathologiestoo promote a better oral condition [9]. In the present case, we report the occurrence of natal teeth in an infant with congenital septic arthritis of the left knee joint. 

Septic arthritis is a fairly uncommon, but serious disorder in newborns. It is characterized by bacterial invasion of the joint space followed by an inflammatory response. The peak incidence is in children younger than three years, and boys are affected approximately twice as often as girls. *Staphylococcus aureus* is the most common causative organism. Symptoms include edema, erythema, joint effusion, and tenderness. Systemic symptoms such as fever, malaise, and poor appetite are also seen in most patients [10].

In the present case, the presence of natal teeth could be attributed to the neonatal sepsis presented in the form of arthritis as well as the presence of a febrile state in the infant due to the sepsis which may have accelerated the tooth eruption. It could also be due to the bone infection which may be related to some type of mineralization defect during the developing period of the fetus and may have led to the superficial positioning of the tooth germ. In addition, the preterm birth of the infant may be a causative factor.

All these factors may have contributed to the accelerated tooth eruption which needs further investigation. Therefore, the dilemma of the correlation of natal teeth with various systemic conditions still needs to be investigated especially as a definite causality cannot be established due to the presentation of these teeth with varying clinical conditions and rarity of occurrence.

## Conclusions

The present report suggests the need for research into neonatal septic conditions as well as febrile illnesses in infants that may predispose them to dental anomalies. The importance of a proper referral system is also highlighted as pediatricians/neonatologists usually are the first to find these teeth, ann early consultation with the dentist could prevent complications. Thus, a multidisciplinary approach and proper evaluation of the child in the first few months of life by the pediatrician/neonatologist and the pediatric dentist can have a long-term effect on the growing child’s overall health and beautiful smile.
